# Comparative agreement between tuberculin skin test and QuantiFERON-TB Gold In-Tube across different levels of exposure in a college-based tuberculosis outbreak

**DOI:** 10.3389/fpubh.2026.1742551

**Published:** 2026-05-12

**Authors:** Zheng Sun, Yunliang Wu, Xiaoyan Ding, Qiao Liu, Yan Shao, Haiqing Zhang, Wei Lu, Limei Zhu, Cheng Chen, Leonardo Martinez

**Affiliations:** 1 Department of Chronic Communicable Disease, Center for Disease Control and Prevention of Jiangsu Province, Nanjing, China; 2 Department of Epidemiology, School of Public Health, Nanjing Medical University, Nanjing, China; 3 Center for Disease Control and Prevention of Xuzhou City, Xuzhou, China; 4 Department of Epidemiology, School of Public Health, Boston University, Boston, MA, United States

**Keywords:** close contacts, IGRAs, latent tuberculosis infection, TST, tuberculosis

## Abstract

**Background:**

Optimal intervention thresholds for students in college TB outbreaks remain debated. Tuberculosis preventive treatment (TPT) is recommended for tuberculin skin test (TST) induration greater than 15 mm. The possibility of TPT recommendation for individuals with TST medium or weak positivity in the context of recent exposure, particularly with close contact with TB, is not entirely clear.

**Methods:**

The study used a cross-sectional design and included a 1-year follow-up of participants after preventive treatment. TST and QuantiFERON-TB Gold In-Tube test (QFT) were used among close contacts of the tuberculosis cases from a college. Since individuals with a strongly positive TST result undergo TPT directly, close contacts with a TST less than 15 mm were further assayed with QFT. TST and QFT were compared based on TB exposure, which was classified as inside or outside classes with TB cases. Student’s *t*-tests, Chi-square tests, Kappa statistics for agreement, and the Cochran–Armitage test for trend analysis were performed.

**Results:**

A total of 1,182 close contacts of TB cases during an outbreak were administered TST. A total of 175 (14.8%, 175/1,182) students had a strongly positive test result (≥15 mm). QFT was performed on the remaining 1,007 students, of whom 85 (8.4%, 85/1,007) tested positive. Among individuals with TST negative indurations (<5 mm), low positivity (5–9 mm), and medium positivity (10–14 mm), QFT positivity was 2.9% (7/237), 5.4% (3/55), and 10.5% (75/715), respectively. The rates of QFT positivity varied with increased TST levels (*χ*^2^ = 13.751, *p =* 0.001). The latent tuberculosis infection rate, as determined by QFT, was significantly higher among students in classes with TB cases (14.11%, 24/149) than among those in classes without TB cases (7.11%, 61/858; *p* < 0.001). Notably, in classes with TB cases, TST and QFT demonstrated moderate agreement (Kappa = 0.52, *p* < 0.001), supporting the use of the 10 mm cutoff to represent infection status in high-exposure settings.

**Conclusion:**

TST showed better concordance with QFT when close contacts were under intense and frequent TB exposure, and a 10 mm result for TST would be a potential recommendation for preventive treatment in close contacts with high exposure to TB transmission.

## Introduction

Tuberculosis (TB) remains a significant global health problem, and it was estimated that 10.8 million new TB cases emerged in 2023, and China accounted for 6.8% of the total cases (1). In high-burden settings, the transmission of *Mycobacterium tuberculosis* is driving the epidemic, leading to the new incidence of TB cases.

Latent tuberculosis infection (LTBI) is defined as a state of persistent immune response to stimulation with *Mycobacterium tuberculosis* antigens, without the evidence of clinically active TB disease (2). Managing LTBI is vital for TB elimination, as approximately 5–10% of individuals with LTBI will eventually develop active TB. The risk is significantly higher among those with recent infections, with the majority of progression occurring within the first 2 years of exposure (3).

According to the WHO, preventive treatment for LTBI includes BCG vaccination. A TST result with induration larger than 15 mm is considered positive (4), and in such cases, tuberculosis preventive treatment (TPT) is recommended. However, the necessity and efficacy of TPT for individuals with medium and weak TST responses (5) remain a subject of debate, particularly in the context of recent close contact with TB.

School outbreaks of TB would be a good simulation of the recent transmission of TB. Effective control in these congregate settings remains a critical public health priority globally, regardless of whether the national TB incidence is high (6, 7) or low (8, 9). However, a significant gap persists regarding the optimal intervention threshold in such high-density environments. To address these gaps, we conducted a cross-sectional investigation to screen close contacts during a college TB outbreak using both TST and IGRAs. This study aimed to evaluate the diagnostic agreement between the two methods across varying levels of tuberculosis exposure and to assess the risk of progression to active TB through a 1-year follow-up of infected contacts.

## Methods

### TB cases and close contacts

**Figure 1:**
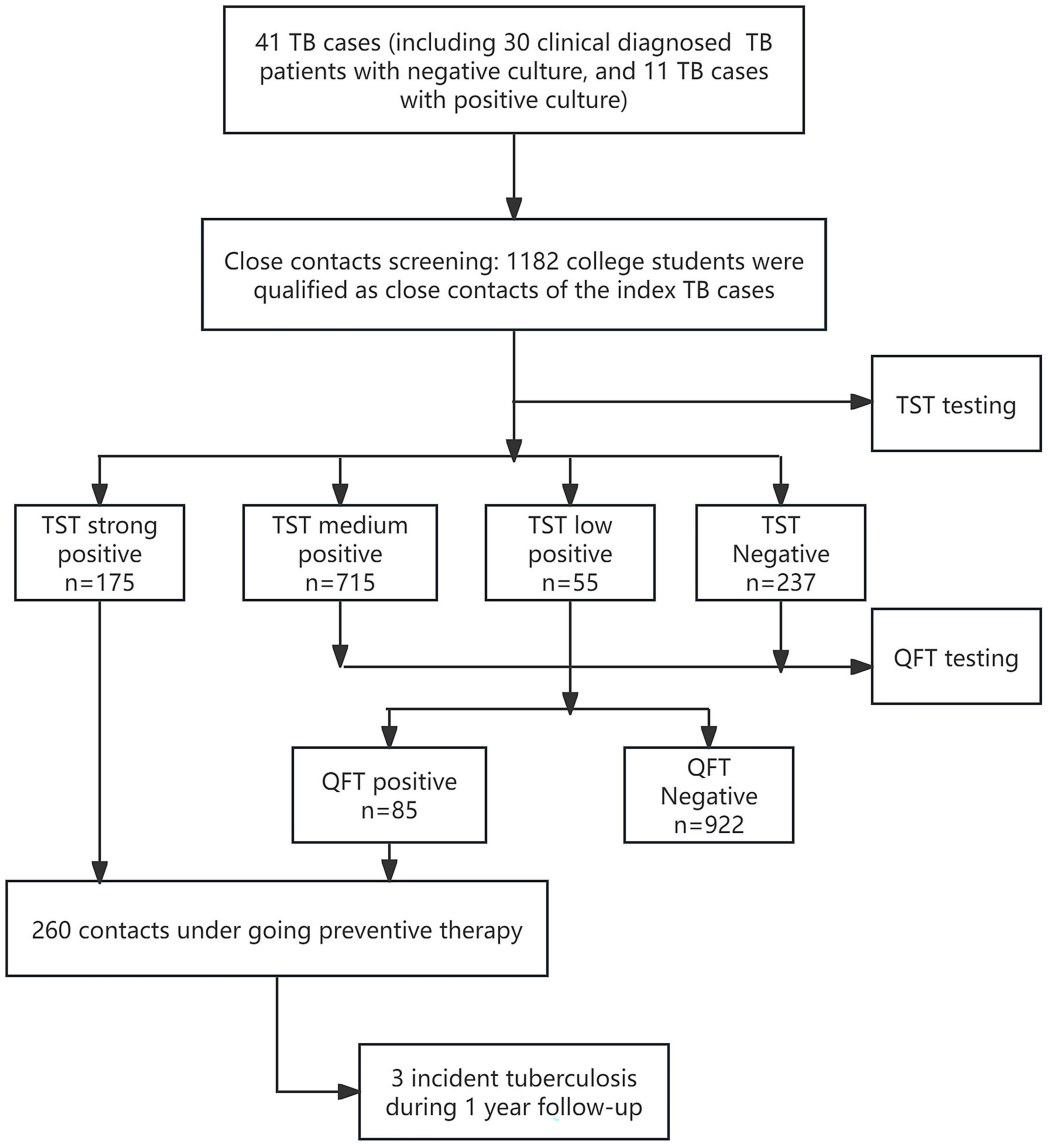
The flowchart of the screening and follow-up of the close contacts of TB cases in an outbreak at a college. TST, tuberculin skin test; QFT, the QuantiFERON-TB Gold In-Tube.

In December 2020, a TB outbreak occurred in a college in Jiangsu province. A total of 41 tuberculosis cases were found during the outbreak, including 11 microbiologically positive TB cases and 30 clinically diagnosed TB cases, and all 11 microbiologically positive TB cases were susceptible to Rifampicin. Close contacts were selected as students who spent at least 8 consecutive hours, or at least 40 cumulative hours (10), with the TB cases since the symptoms of TB occurred. Chest X-rays were provided to all close contacts, and those with abnormal X-rays were provided with microbiological tests, including smear sputum, and GeneXpert MTB/RIF. We conducted a cross-sectional study by screening for LTBI status for the close contacts with normal X-ray results. Contacts with a previous history of TB were not included in LTBI screening (Figure 1).

### Screening stages for close contacts

In this study, BCG-PPD at 50 IU/mL for 10 tests was used for the tuberculin skin test (TST) [BCG-PPD, Simcere, Beijing, China], and the QuantiFERON-TB Gold In-Tube test [QFT; Qiagen, Valencia, CA, USA] was administered to evaluate *Mycobacterium tuberculosis* infection. QFT contained three tubes for the assay: the Nil tube, the mitogen tube, and the TB antigen tube. The procedures for both tests were described in the previous study (11).

In the first phase, TST was performed on all close contacts. TST results were categorized into low, medium, and strong positivity based on the size of the millimeter induration reaction. Strong positivity was defined as an induration diameter ≥ 15 mm, and double circles, blisters, necrosis, or local lymphangitis were also considered strong positivity (12) and were classified as the high-risk group. The medium positivity of TST was defined as an induration diameter of 10–14 mm. Low positivity was defined as an induration diameter of 5–9 mm. Students with indurations <5 mm were considered negative (5). During the second screening phase, students with medium or low-positive TST were retested with QFT. In this study, QFT was used as the comparative immunologic test for LTBI detection, acknowledging the absence of a true gold standard (13). We defined TST < 10 mm as the non-infection group and TST > 10 mm and <15 mm as the infection group. To further evaluate the impact of exposure intensity on diagnostic performance, close contacts were categorized into two groups: those sharing a classroom with an index TB case and those from classes without an index case. This analysis aimed to assess whether diagnostic agreement between TST and QFT increases with the likelihood of infection. Students with strongly positive TST results and those with a positive QFT result (during secondary testing) were offered 3HR preventive chemotherapy according to the Guidelines for tuberculosis prevention and control in Chinese schools. Students were followed for 1 year after preventive therapy; during follow-up, they were screened for tuberculosis-related symptoms and underwent an X-ray examination. Written informed consent was obtained from all participants during the tuberculosis outbreak. This study was approved by the institutional review board of the Jiangsu Provincial Center for Disease Control and Prevention. Written informed consent was obtained from each participant before data collection.

### Statistical analysis

Variables in normal distribution are described using mean and standard deviation, and median with interquartile range are used to describe variables with non-normal distribution. The Student’s *t*-test or Mann–Whitney *U*-test was used to compare the contentious values of the basic characteristics of the close contacts and the values of QFT TB antigen depending on the variable distribution. The chi-square test was used to compare the categorical variables. Kappa was calculated to indicate the strength of agreement: 0–0.20, poor and slight; 0.21–0.40, fair; 0.41–0.60, moderate; 0.61–0.80, substantial; and 0.81–1, almost perfect (14). The Cochran–Armitage method was adopted for the *P* trend test. *p*-values less than 0.05 were considered statistically significant. All analyses were performed using SAS 9.3 software (SAS Institute, Inc., Cary, NC, USA).

## Results

During the outbreak, 1,182 close contacts underwent TST screening. The TST was performed on all students, and the 175 students (14.8%) who exhibited strong positivity were not tested with QFT. Then, 1,007 individuals were given the QFT test. The QFT assay showed 85 (8.4%, 85/1007) individuals had a positive result. The median age between QFT-positive and QFT-negative individuals showed no significant difference (*p =* 0.203). The QFT positivity among males was 10.3, and 7.5% among females, and the difference showed no statistical significance (*χ*^2^ = 2.346. *p =* 0.126). Among persons with negative TST results, QFT positivity was 2.9% (7/237); for low positive TST, the QFT positivity was 5.4%; and for medium positive TST, the QFT positivity was 10.5%. The QFT positive rates differed significantly across TST levels (*χ*^2^ = 13.751, *p =* 0.001). The Cochran–Armitage trend test confirmed a significant increase in QFT positivity with advancing TST levels (*p =* 0.0002). In the next step, we defined TST less than 10 mm as the non-infection group and those at least 10 mm and less than 15 mm as the infection group. We found the comparison between QFT and TST reached statistical significance (*χ*^2^ = 13.390, *p* < 0.001), and the Kappa value was 0.043, showing an extremely low agreement between the two methods (Table 1).

**Table 1:** Comparison of the tuberculin skin test and QuantiFERON-TB Gold In-Tube for close contacts of TB.

Characteristics	QuantiFERON-TB Gold In-Tube		Total	*χ* ^2^	*p*
	Negative	Positive			
*N*	922	85	1,007		
Age (median)	20.0	20.0	20.0		0.203^§^
Gender				2.346	0.126
Male	304 (89.7)	35 (10.3)	339		
Female	618 (92.5)	50 (7.5)	668		
Tuberculin skin test (induration)				13.75	0.001
Negative (<5 mm)	230 (97.1)	7 (2.9)	237		
Low positive (5–9 mm)	52 (94.6)	3 (5.4)	55		
Medium positive (10–14 mm)	640 (89.5)	75 (10.5)	715		0.0002^**^
Tuberculin skin test (infection/non-infection)^*^				13.39	<0.001
Non-infection	282 (96.6)	10 (3.4)	292		
Infection	640 (89.5)	75 (10.5)	715		

TST, tuberculin skin test; QFT, the QuantiFERON-TB Gold In-Tube; TB, tuberculosis.

^*^TST induration of ≥10 mm was considered infection, and those less than 10 mm were considered non-infection. Kappa value was 0.043.

^**^Comparison of QFT positive rates among the negative, low positive, and medium positive groups of TST by the Cochran–Armitage trend test.

^§^Mann–Whitney *U*-test.

**Figure 2:**
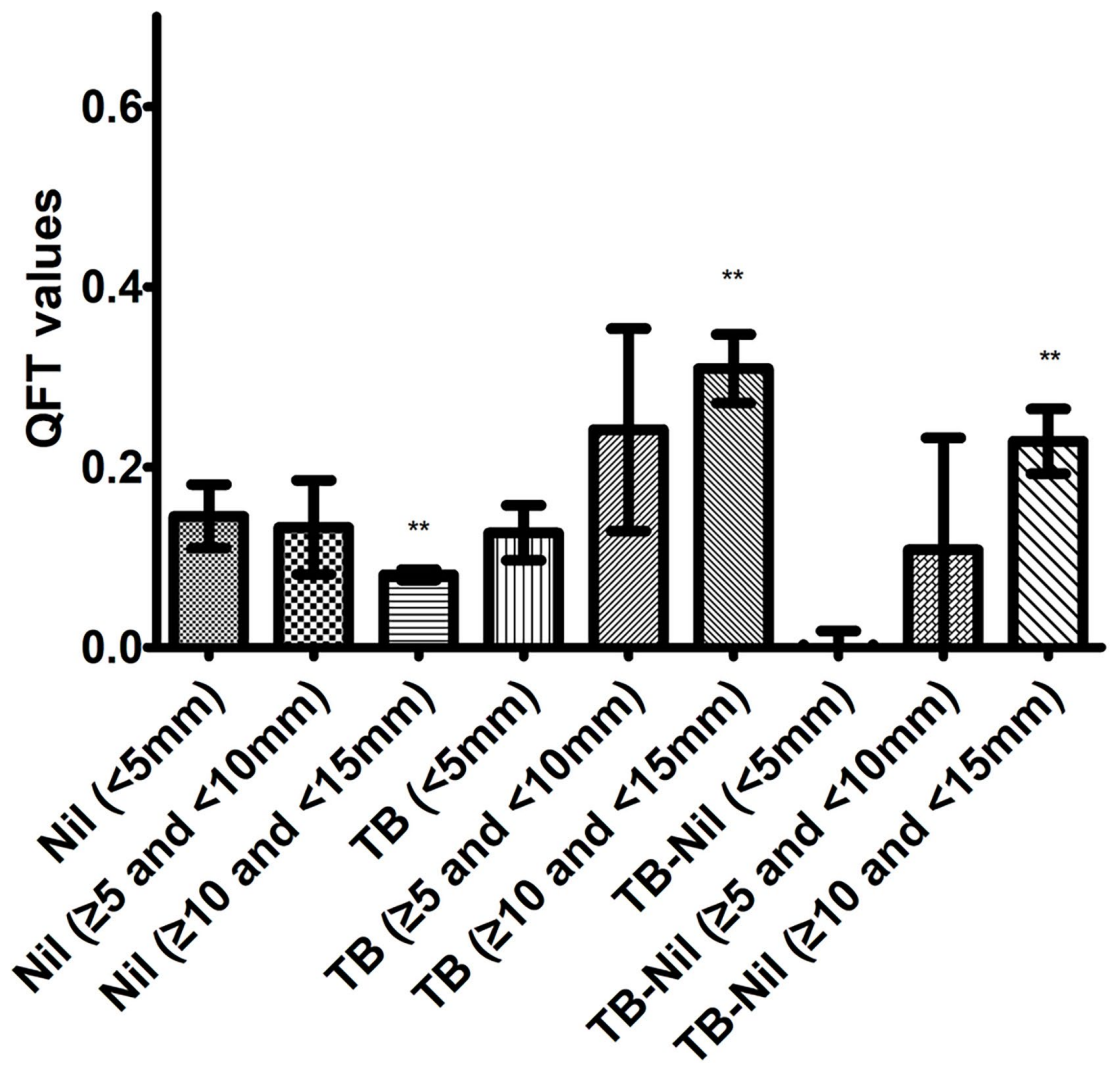
Comparison of QFT Nil and TB-antigen stimulated IFN-*γ* concentration among different TST categories. A total of 1,008 close contacts were assayed by QFT, with 82 positive and 922 negative, ^**^ represents *p* < 0.05. TST, tuberculin skin test. QFT, the QuantiFERON-TB Gold In-Tube.

We further compared the mean concentrations of the QFT Nil control and TB antigens across different TST reactivity levels (Figure 2). For the QFT TB-antigen stimulated IFN-*γ* concentration, we found it was significantly high in the TST medium positive group (0.31 ± 0.04) when compared to the TST negative group (0.13 ± 0.03, *p =* 0.008), and the TB-antigen stimulated IFN-γ concentration was high in the low positive TST group but reached no significant difference when compared to the TST negative group (0.24 ± 0.11, *p =* 0.171). Meanwhile, the values of TB antigen stimulated IFN-γ concentration minus Nil control were also significantly higher in the TST medium positive group (0.23 ± 0.04) when compared to the negative result of TST (−0.02 ± 0.04, *p* < 0.001), but TB antigen stimulated IFN-γ concentration minus Nil control in the low positive TST group (0.11 ± 0.12) was only high compared to TST negative group and reached no significant difference (*p =* 0.191).

Close contacts were categorized into two groups based on whether their classroom included index TB cases to analyze the impact of exposure intensity on QFT positivity (Table 2). QFT positivity for close contacts with TB cases in class (16.1%, 24/149) was significantly higher than that from class without TB cases (7.1%, 61/858, *χ*^2^ = 13.30, *p* < 0.001). TST indurations and QFT TB-Nil values for QFT positive close contacts in and out of TB class showed no significant difference (*p* = 0.16, and *p* = 0.63).

**Table 2:** Comparison of TST indurations and QFT TB-antigen stimulated IFN-γ concentration among QFT positive close contacts in and out of TB classes.

All classes	Latent tuberculosis Infection Assay methods		
	QFT positivity *N* (%)	TST induration (mm)	QFT TB antigen-nil (IU/mL)
Classes without case (*n* = 858)	61 (7.11%)	10.74 ± 2.88	2.33 ± 2.20
Classes with case (*n* = 149)	24 (16.11%)	10.33 ± 4.97	1.64 ± 1.69
	*χ*^2^ = 13.30, *p* < 0.001	*F* = 1.95, *p =* 0.16	*F* = 0.23, *p =* 0.63

TST, tuberculin skin test; QFT, the QuantiFERON-TB Gold In-Tube; TB, tuberculosis.

Upon evaluating the agreement of TST and QFT results among classes with tuberculosis cases, kappa values were 0.52 (95%CI, 0.34–0.69) and demonstrated a moderate agreement between TST and QFT (*p* < 0.001). The PPV was 50% (95%CI, 38.65–61.35), and the NPV was 95.5% (95%CI, 90.64–97.89) for the TST method compared to QFT (Table 3).

**Table 3:** Paired analysis of QuantiFERON Gold In-Tube and tuberculin skin test results among close contacts in classrooms of TB cases.

Class #	TB cases/all students (%)	Contacts	TST^*^	QFT positive (%)	Kappa (95%CI)	PPV, %(95%CI)	NPV, %(95%CI)	*p*
Class 1	1/45 (2.2%)	35	Negative	1 (2.9)				
		9	Positive	1 (11.1)				
Total		44		2 (4.5)	0.12 (0–0.63)	11.11 (2.66–36.36)	97.14 (89.40–99.28)	0.289
Class 2	29/44 (65.9%)	2	Negative	2 (100.0)				
		13	Positive	11 (84.6)				
Total		15		13 (86.7)	−0.15 (/)	84.62 (81.35–87.40)	0 (/)	0.551
Class 3	11/54 (20.4%)	31	Negative	1 (3.3)				
		12	Positive	5 (41.7)				
Total		43		6 (14.0)	0.45 (0.11–0.79)	41.67 (25.10–60.36)	96.77 (83.27–99.45)	0.001
Class 4	1/48 (2.1%)	43	Negative	1 (2.3)				
		4	Positive	2 (50.0)				
Total		47		3 (6.0)	0.54 (0.03–1.00)	50.00 (17.18–82.82)	97.77 (89.44–99.52)	<0.001
All	42/191 (22.0%)	111	Negative	5 (4.5)				
		38	Positive	19 (50.0)				
Total		149		24 (16.1)	0.52 (0.34–0.69)	50 (38.65–61.35)	95.50 (90.64–97.89)	<0.001

^*^TST induration of ≥10 mm was considered positive, and those less than 10 mm were negative.

PPV: positive predictive value; NPV: negative predictive value.

All persons with either TST strong positivity or IGRA positivity were prescribed a daily isoniazid and rifampicin regimen for 3 months, according to the WHO guidelines. After 1-year follow-up of the close contacts after the preventive therapy, three contacts (1.15%, 3/260) developed TB disease, including one person in the TST strong positive group (1/175, 0.57%) and the other two in the QFT positive group (2/85, 2.35%), and the corresponding TST result was in the medium positivity (2/715). Meanwhile, no TB cases were reported among contacts with negative QFT results (0/922; 0%).

## Discussion

In this college TB outbreak, 1,182 close contacts were screened for TB disease and *Mycobacterium tuberculosis* infection. TST results showed an overall positivity (>5 mm induration) of 80%. However, the majority of these contacts did not have confirmatory positive QFT results, suggesting high false-positive rates with TST and poor concordance between TST and QFT. Notably, while QFT results demonstrated a higher rate of LTBI among TB classes than the outside classes contacts, and QFT TB-antigen stimulated IFN-*γ* concentration for those LTBI in and outside the classes showed no significant difference, which implied interferon γ levels for the positive QFT would not be linearly increased by much more frequent contacting to the TB, and the current close contact definition was adequate to find out those potential LTBI. After a 1-year follow-up of the screening population, three persons developed incident TB, which suggested that TST in combination with IGRA would be a good strategy for LTBI detection in school outbreaks, and a TST result of 10 mm or above for the definition of TST positivity would be acceptable for LTBI screening.

A prior study that used TST and IGRAs during a school tuberculosis outbreak (15) found that IGRA was an essential addition to *Mycobacterium tuberculosis* screening in areas with high coverage of BCG vaccination. IGRAs were more likely to predict incident tuberculosis risks than the tuberculin skin test. However, TST, combined with IGRAs, may be more suitable for identifying target populations for whom preventive therapy is more effective.

A previous meta-analysis showed that the prevalence of LTBI in school outbreaks in mainland China ranged from 19.5 to 28.9% (16), slightly higher than our results. Previously, we conducted a cross-sectional study of LTBI screening in the general population, which showed that the prevalence of LTBI was only 20%, and LTBI rates in the age groups between 10 and 29 years ranged from 6.9 to 8.8% (17). In this study, the LTBI rate was 8.4% by QFT. The LTBI rate by QFT was lower than that of the general population in this study, but if we included those with strongly positive TST, the LTBI would increase for this age group. Meanwhile, we found a gradual increase in the QFT rate from TST low-positive contacts to medium-positive contacts. Previous studies indicated a high concordance between strong positive TST and positive IGRA results (18). Thus, close contacts with a strongly positive TST were not included in the QFT assay according to the screening strategy of the study.

WHO recommends preventive treatment for individuals with TST results of 10 mm or greater (19). In our study, 10 mm was also used as the cutoff for TST to determine positive results for LTBI; approximately 90% of TST-positive results were false positives when compared with QFT-negative results, indicating a high discrepancy between the two assays. A previous study evaluated TST and QFT concordance (11) and found that 60.11% of QFT-negative results were among TST-positive individuals. The high coverage of BCG vaccination could explain the high false-positive rate of TST among these teenagers. TST uses purified protein derivative (PPD), a mixture of antigens shared by both the BCG vaccine and *Mycobacterium tuberculosis*, leading to high false-positive rates due to antigenic cross-reactivity. In contrast, QFT uses specific antigens (ESAT-6 and CFP-10) encoded in the RD1 region, which is present in *Mycobacterium tuberculosis* but deleted from all BCG vaccine strains. Consequently, QFT is unaffected by prior vaccination. Although the TST and QFT results showed a high discrepancy, the QFT positivity rate increased gradually with higher TST positivity levels, and the majority of QFT-positive results were among participants with TST results between 10 and 15 mm (88%). Our findings are consistent with the WHO-recommended standards.

A previous study conducted by Cao et al. found that TST for at least 10 mm increased the risk of developing TB in college students (7). Martinez et al. conducted a systematic review that revealed that children with TST induration above 10 mm not only increased the risk of prevalent TB but also the risk of incident TB (20). Our study found that all three incident TB cases were in the medium- to high-positive TST groups. Although those three LTBI persons finished 3 months of preventive treatment, TB disease finally developed. Even though TPT had a strong protective effect on LTBI, a small proportion would develop TB (21). Additionally, despite the cases exhibiting sensitivity to rifampin, individual variations in drug metabolism or fluctuations in medication adherence in non-hospital settings may have compromised the preventive efficacy. However, no incident TB cases occurred in those with negative QFT. All three incident TB cases occurred in the first year of follow-up, as a previous study indicating that over 90% of close contacts developed TB within 12 months of TB exposure (22). The 10 mm of TST was a significant cutoff for recommending preventive treatment for LTBI.

We further analyzed the QFT TB-antigen and Nil in relationship with different categories of TST, and we found that the values of QFT TB-antigen and TB-antigen minus Nil were significantly higher in the TST medium-positive group than in the TST negative group. However, the QFT Nil and TB-antigen values did not differ significantly between the TST-negative and low-positive groups. Our results demonstrated that QFT TB-antigen values did not differ by more than 10 mm on TST and provided evidence for adopting 10 mm as a reasonable cutoff value for TST in determining LTBI status in recent transmission.

We further analyzed the relationship between the levels of QFT TB-antigen and the degree of exposure to TB cases. We found that the level of QFT TB-antigen-Nil was not associated with the frequency of close contact with TB cases.

Currently, there is no suitable method to monitor the treatment effect of LTBI; a previous study found that QFT is unlikely to be a valuable biomarker of response to preventive treatment (23, 24). Meanwhile, neither IGRA nor the TST has high accuracy for predicting active tuberculosis (25). Transcriptomics (26) and proteomics have made some progress in this regard (27). Further research is required to validate these findings.

There are several limitations to this study. First, according to the screening strategy, those individuals with strong positivity of TST were not retested with QFT, and we cannot evaluate QFTTB-antigen stimulated IFN-*γ* levels in the TST strong positivity group. Second, follow-up of close contacts was only 1 year after the outbreak; potential incident TB cases might emerge with prolonged follow-up. However, according to the recommendations of the Chinese anti-TB association, those with a strong positive TST need to take preventive therapy without considering the IGRA results.

In conclusion, our study revealed that 10 mm is likely to be an acceptable cutoff value for TST when screening close contacts in a TB outbreak, especially for individuals who had frequently contacted with the TB cases; QFT positivity was increased by much more frequent contact with the TB cases, but TST induration or QFT TB-antigen stimulated IFN-γ concentration was not greatly increased with frequent contacting with the TB for positive QFT contacts.

## Data Availability

The raw data supporting the conclusions of this article will be made available by the authors, without undue reservation.
